# Molecular diagnosis of an infant with *TSC2*/*PKD1* contiguous gene syndrome

**DOI:** 10.1038/s41439-020-0108-0

**Published:** 2020-07-16

**Authors:** Keita Osumi, Kenichi Suga, Akemi Ono, Aya Goji, Tatsuo Mori, Yukiko Kinoshita, Mikio Sugano, Yoshihiro Toda, Maki Urushihara, Ryuji Nakagawa, Yasunobu Hayabuchi, Issei Imoto, Shoji Kagami

**Affiliations:** 1 Department of Pediatrics, Tokushima University Hospital, Kuramotocho, Tokushima, Tokushima Japan; 2 Department of Cardiovascular Surgery, Tokushima University Hospital, Kuramotocho, Tokushima, Tokushima, Japan; 3 Department of Preventive Medicine, Division of Molecular Genetics, Aichi Cancer Center Research Institute, Nagoya, Aichi Japan; 4 Department of Cancer Genetics, Nagoya University Graduate School of Medicine, Nagoya, 466-8550 Japan

**Keywords:** Polycystic kidney disease, Genetic testing

## Abstract

A 1-month-old Japanese infant with cardiac rhabdomyoma was diagnosed with *TSC2*/*PKD1* contiguous gene syndrome by targeted panel sequencing with subsequent quantitative polymerase chain reaction that revealed gross monoallelic deletion, including parts of two genes: exons 19–42 of *TSC2* and exons 2–46 of *PKD1*. Early molecular diagnosis can help to detect bilateral renal cyst formation and multidisciplinary follow-up of this multisystem disease.

Tuberous sclerosis complex (TSC, OMIM #191100 and #613254) is an autosomal dominant multiple system disorder characterized by hamartomatous growth abnormalities in multiple organ systems, including the brain, skin, heart, lungs, and kidneys^1^. TSC has a broad phenotypic spectrum, including seizures, mental retardation, renal dysfunction, and dermatological abnormalities and tumors, and the clinical manifestations of TSC vary individually. TSC is caused by pathogenic variants in either one of the two disease-causing genes, *TSC1* (OMIM #605284, 9p34) or *TSC2* (OMIM #191092, 16p13.3), encoding hamartin and tuberin, respectively^2^. Pathogenic variants in *TSC2* occur 4–5 times more often and are associated with a more severe phenotype than those in *TSC1*^1^. The *TSC2* gene lies immediately adjacent to *PKD1* (OMIM #601313), which encodes polycystin-1 and is the major gene causing autosomal dominant polycystic kidney disease (ADPKD, MIM#173900). ADPKD is characterized by progressive bilateral renal cysts and is sometimes complicated by liver cysts and intracranial aneurysms. Both genes are in tail-to-tail orientation. Large deletions disrupting both *TSC2* and *PKD1* result in *TSC2/PKD1* contiguous gene syndrome (PKDTS, MIM#600273). This disease was first reported by Brook–Carter et al. in 1994 with a variety of phenotypes dominated by severe, very early-onset PKD and occurs in ~2–5% of TSC patients, resulting in significant renal insufficiency in teenage years^3–8^.

We present herein the case of a male infant with TSC in whom multiple cardiac rhabdomyomas were detected at 27 weeks of gestation on ultrasound. Retinal hamartoma, multiple subependymal nodules, cortical tubers, and bilateral multiple renal cysts were detected after birth. Targeted panel sequencing (TPS) identified the gross germline deletion involving parts of the *TSC2* and *PKD1* genes, which has never been reported before.

The male infant was born as the first child of healthy, nonconsanguineous Japanese parents with an unremarkable family history. At 27 weeks of gestation, cardiac tumors were detected on fetal ultrasonography, and a massive tumor was seen protruding from the ventricular septum to the left ventricle with multiple small tumors on both ventricle walls (Fig. 1a). Fetal ultrasound and magnetic resonance imaging also showed a 2 cm diameter tumor with a mixture of high and low echo areas and increased and decreased signal intensity areas, respectively, in the right kidney. He was delivered at 37 weeks and 5 days of gestation by vaginal delivery. At birth, the weight was 3468 × *g* (+2.2 SD), length was 53 cm (+2.9 SD), and head circumference was 36 cm (+2.4 SD) with no marked physical presentation. Retinal hamartoma was observed as an ocular lesion near the macula of the right eye. On neonatal ultrasonography, no clear renal cysts were observed, although a tumor was detected in the lower pole of the right kidney. Because the obstruction of the left ventricular outlet by the tumor gradually worsened, the cardiac tumor underwent partial surgical resection when he was 29 days old. Pathological findings for the tumor were compatible with rhabdomyoma, and residual tumors regressed spontaneously. Magnetic resonance imaging showed multiple bilateral renal cysts as well as multiple subependymal nodules and cortical tubers (Fig. 1b–d). Although seizures were not evident clinically, electroencephalography at 5 months old showed paroxysmal focal spikes in the right frontal region, resulting in the initiation of valproate treatment. Based on these findings, TSC was clinically suspected, whereas PKD was not suspected at that time^9^. The diagnosis of PKDTS was genetically confirmed by applying TPS for a combined screening of causative alterations in candidate genes as described below at 2 months old. As of the time of writing, at 3 years old, he was healthy with normal renal function, no progression of the ocular lesion, and normal neurological development without epileptic attacks.

**Fig. 1:**
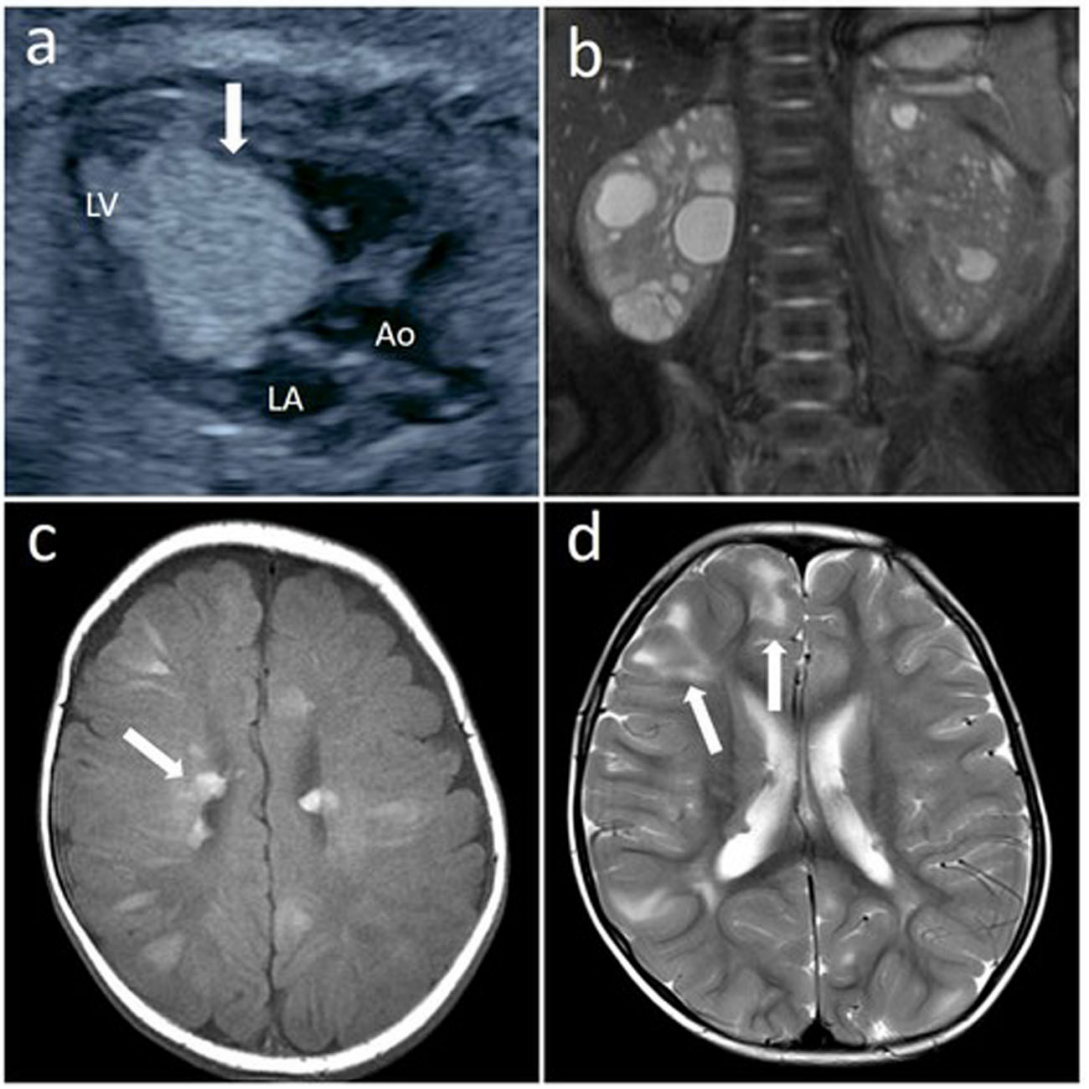
Representative image findings. **a** Fetal echocardiography showing rhabdomyoma (white arrows), Ao aorta, LA left atrium, LV left ventricle. **b** Magnetic resonance imaging (MRI, T2-weighted image) of abdomen at 3 months old showing enlarged kidneys with multiple cysts. **c** MRI (T1-weighted image) of brain at 55 days old showing multiple subependymal nodules along the lateral ventricle walls (white arrows). **d** MRI (T2-weighted image) of brain at 3 years old showing multiple subcortical high signal lesions indicating cortical tubes in cerebral hemisphere, predominantly in frontal lobe (white arrows).

The ethics committee at Tokushima University approved this study. After written informed consent was obtained from the parents of the patient, genetic analysis was performed using genomic DNA extracted from whole blood obtained from the patient at 2 months old. We conducted TPS for the exons of 4813 disease-related genes using the Trusight One Sequencing Panel (Illumina, San Diego, CA) and a MiSeq sequencer (Illumina), followed by analysis using our pipeline for next-generation sequencing (NGS) data, as described previously^10–12^. GRCh37/hg19 (build 37 of the Genome Reference Consortium human genome) was used as the human reference genome sequence. Detection of copy number variations (CNVs) using TPS data with resolution ranging from a single exon to several exons depending on exon size was performed as described previously^11^. These analyses showed no disease-causing SNVs or small insertions/deletions (indels) but detected a gross monoallelic deletion at least 47.6 kb long, including parts of two genes: exons 19–42 of *TSC2* (NM_000548.5) and exons 2–46 of *PKD1* (NM_001009944.3), located from positions 2,121,775–2,169,389 of chromosome 16 (16p13.3), suggesting *TSC2*/*PKD1* contiguous gene syndrome as a molecular diagnosis (Fig. 2a). However, the data from the probe for exon 1 of *PKD1* for TPS were not available, possibly due to a relatively high GC content. To validate a deletion and determine deletion breakpoints, we performed a SYBR green-based real-time quantitative polymerase chain reaction (qPCR) as described elsewhere (Fig. 2a, Supplementary table S1)^13^. Monoallelic deletions of parts of *TSC2* and *PKD1* were validated, and the breakpoints appeared to be located between exons 18 and 19 of *TSC2* and between exons 1 and 2 of *PKD1* (Fig. 2b), suggesting that the maximum size of the deleted region is ~64 kb. The deletion was not confirmed as de novo because of the unavailability of parental DNA.

**Fig. 2:**
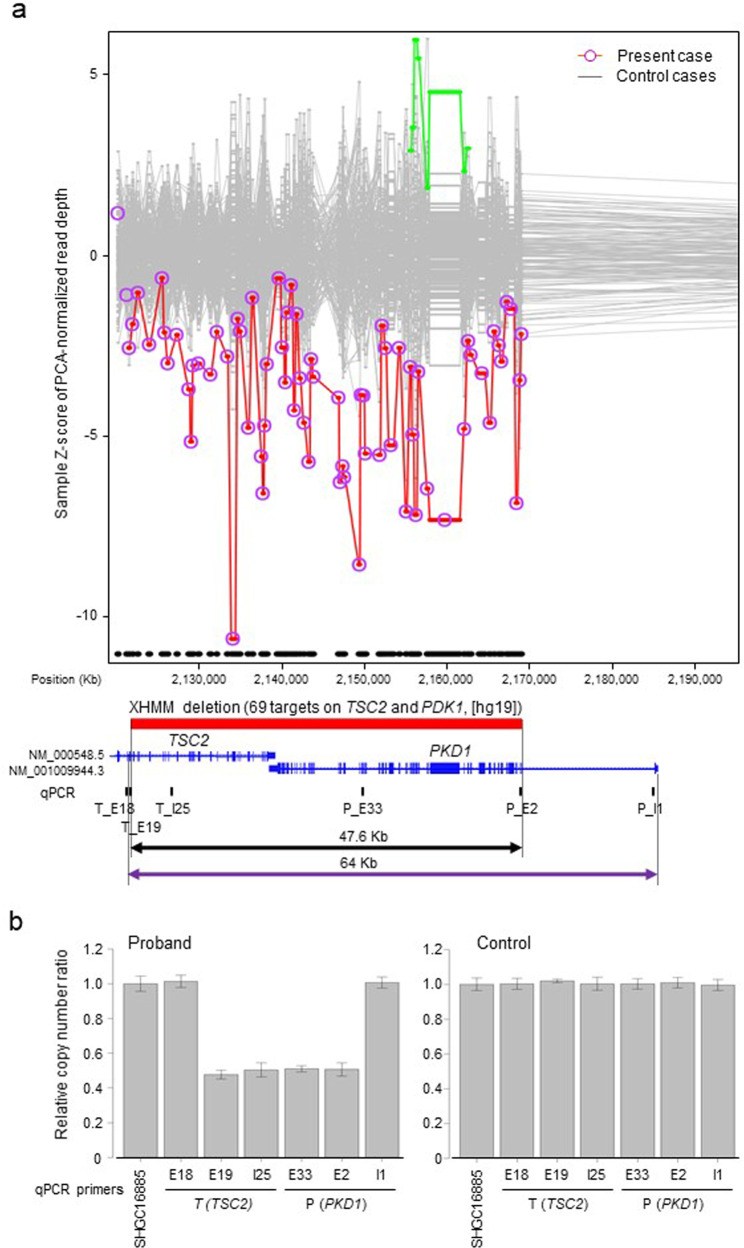
Genetic analysis. **a** An eXome-Hidden Markov Model v1.0 (XHMM, https://atgu.mgh.harvard.edu/xhmm/) analysis using TPS data detected a gross monoallelic deletion at least 47.6kb long (black closed arrow), including parts of two genes: exons 19–42 of *TSC2* and exons 2–46 of *PKD1*, located from positions 2,121,775–2,169,389 of chromosome 16 (red solid bar). The *x*-axis denotes the physical position, and the *y*-axis indicates the Z-score of principal component analysis (PCA)-normalized read depth. Purple circles connected by red lines show values for the individual subjected to TPS in this study. Gray dots with gray connected lines represent the results of normalized read depth obtained from in-house control data (*N*=126). Blue vertical bars in each gene represent exons. Because the data from the probe for exon 1 of *PKD1* for TPS were not available, we could not determine the break point around exon 1 of *PKD1* by TPS, although the break point within *TSC2* appeared to be located between exons 18 and 19. To validate a deletion and determine deletion breakpoints, we performed SYBR green-based qPCR using primer sets targeted to six sites around two genes (black vertical bars) and the control site, SHGC16885 (Supplementary table S1). **b** Results of qPCR to determine a relative genomic copy number using genomic DNA from the proband in the present study (left) and the healthy male control (right). Based on the relative copy numbers determined by qPCR, monoallelic deletions of parts of *TSC2* and *PKD1* were validated, and the breakpoints appeared to be located between exons 18 and 19 of *TSC2* and between exons 1 and 2 of *PKD1*, suggesting that the maximum size of the deleted region is ~64kb (purple closed arrow).

According to the Human Gene Mutation Database professional 2020.1 (HGMD, https://portal.biobase-international.com/hgmd/pro/all.php), 1227 damaging variants have been reported in *TSC2*. Of these, 176 (14.3%) were gross deletions, and 32 (2.6%) involved simultaneous deletion of the adjacent *PKD1* gene, although the number of variants does not directly indicate the prevalence of PKDTS. In addition, heterozygous pathogenic variants in both *TSC1* and *TSC2* are responsible for TSC. Because only TSC was clinically suspected in the present case at birth, we applied TPS based on next-generation sequencing (NGS) technology to simultaneously investigate SNVs, indels and CNVs in most exons of candidate disease-causing genes, *TSC1* and *TSC2*, and their adjacent genes. Our findings demonstrated the utility of NGS for improving the molecular diagnosis of TSC and PKDTS in a timely and cost-effective manner. Previously, both multiplex ligation-dependent probe amplification (MLPA) and array comparative genomic hybridization (array-CGH) were shown to be useful not only for the diagnosis of PKDTS but also for elucidation of its molecular mechanism^14,15^. However, it is also known that a prompt diagnosis of PKDTS can be complicated by the phenotypic heterogeneity of PKD and the absence of a clear phenotype-genotype correlation^15^. Under conditions where there is no strong clinical suspicion of PKDTS, NGS-based technology can be used to simultaneously evaluate multiple genetic alterations causing TSC.

Although ~25–32% of TSC cases exhibit some degree of renal cyst formation, most of which occurs in the second decade of life^4,5^, PKDTS seems typically associated with severe juvenile polycystic kidney disease^3,6–8^. Sampson et al. reported that among 27 unrelated patients with TSC and renal cystic disease, cystic disease was more severe, with early renal insufficiency, in 17 patients with PKDTS showing constitutional deletions^7^. Patients with PKDTS should therefore be diagnosed as early as possible to predict the increased risk of ADPKD-related complications, including cystic kidney disease as well as early end-stage renal disease^6,7,15^. In addition, most patients with TSC also develop neurological and neuropsychiatric disorders, with up to 90% developing epilepsy and up to 50% developing autism, suggesting that treating infants with TSC and epilepsy early with antiepileptic medications or surgery may result in better neurological outcomes^16^. In summary, early molecular diagnosis of PKDTS may be crucial for providing appropriate disease-related surveillance and therapeutic options in patients, as well as appropriate genetic counseling for the family.

## Supplementary information

Supplementary Table S1

## Data Availability

The relevant data from this Data Report are hosted at the Human Genome Variation Database at, 10.6084/m9.figshare.hgv.2870, 10.6084/m9.figshare.hgv.2873.
